# Expression of Estrogen Receptor Coactivator Proline-, Glutamic Acid- and Leucine-Rich Protein 1 within Paraspinal Muscles in Adolescents with Idiopathic Scoliosis

**DOI:** 10.1371/journal.pone.0152286

**Published:** 2016-04-05

**Authors:** Izabela Skibinska, Marek Tomaszewski, Miroslaw Andrusiewicz, Paulina Urbaniak, Roza Czarnecka-Klos, Milud Shadi, Tomasz Kotwicki, Malgorzata Kotwicka

**Affiliations:** 1 Department of Cell Biology, Health Sciences Faculty, University of Medical Sciences, Poznan, Poland; 2 Spine Disorders and Pediatric Orthopedics Department, Faculty of Medicine I, University of Medical Sciences, Poznan, Poland; Roswell Park Cancer Institute, UNITED STATES

## Abstract

**Purpose:**

The aim of this study was to detect and assess the estrogen receptor (ESR) coactivator *PELP1* expression within human paraspinal skeletal muscles in patients suffering from idiopathic scoliosis.

**Methods:**

During surgical correction of scoliosis the muscle biopsies harvested in 29 females. Presence of *PELP1*, *ESR1* and *ESR2* genes transcripts was studied using RT-qPCR technique while immunohistochemistry and western blot methods were used to detect the PEPL1 protein presence.

**Results:**

*PELP1* expression in deep paraspinal muscles revealed higher than in superficial back muscles (p = 0.005). Positive immunohistochemical staining for PELP1 was observed in the nuclei of the paraspinal muscle cells. Western blot revealed PELP1 protein in all samples. No significant difference in *PELP1* expression between the convex and the concave scoliosis side (p>0.05) was found. In deep paraspinal back muscles, a significant correlation between the *PELP1* expression level on the concave side and the Cobb angle (r = 0.4; p<0.05) was noted as well as between the *PELP1* and *ESR1* expression level (r = 0.7; p<0.05) while no correlation between *PELP1* and *ESR2* expression level was found.

**Conclusion:**

To our knowledge, three techniques for the first time demonstrated the presence of the PELP1 in paraspinal muscles of patients with idiopathic scoliosis. The PELP1 potential regulatory impact on back muscle function is to be further investigated.

## Introduction

Almost 80% of scoliosis is considered idiopathic, i.e. of unknown cause. It has been assumed for years that deep paraspinal muscles activity plays a role in the pathogenesis of idiopathic scoliosis (IS) [1]. Functional imbalance of muscles on both sides of the scoliotic curve has been observed [2]. One of the theories on the aetiology of IS is associated with potential influence of estrogens and estrogen receptors on paraspinal muscle cells function [3,4]. Estrogens affect muscular tissue function in terms of adaptation to the endurance training by angiogenesis and miogenesis caused by the estrogen-associated angiogenic factor VEGF (vascular endothelial growth factor) [5] as well as by influencing production of the nitric oxide being the vasodilatator [6]. Moreover, estrogens suppress bone remodeling and control balance between bone formation and resorption. First increase of estrogens blood level appears during puberty and correlates with IS progression [7]. Studies on levels of circulating estrogens in girls with idiopathic scoliosis versus healthy girls are inconsistent [8,9].

Two estrogen receptors (ER) have been identified: estrogen receptor 1 (ESR1) previously called alpha and estrogen receptor 2 (ESR2), called beta. Estrogen receptors belong to nuclear receptors; they present transcriptional activity and were previously identified in skeletal muscle cells [5,10]. The estrogen-ER complex acts through both the classical genomic and the rapid non-genomic signaling pathway. In the classical genomic pathway, the ERs are transcriptional factors that regulate gene expression in a ligand-dependent manner [11]. The transcriptional activity of the ER is regulated not only by estrogens but also by other regulatory proteins called coactivators and corepressors [12]. Proline-, glutamic acid-, and leucine-rich protein 1 (PELP1) is one of important estrogens coactivators. PELP1 plays a crucial role in ESR1 signaling by directly interacting with CARM1 (coactivator-associated arginine methyl-transferase) which synergistically enhances ESR1 activation [13]. Parallel model of PELP1 involvement in ESR2 signaling was reported using the Ishikawa endometrial cancer model cell line and ER subtype-specific ligands [14].

PELP1 protein is relatively recently described as the scaffolding protein that binds various signaling complexes with nuclear receptors and plays a role in either genomic and non-genomic pathways [15]. In the SDS-Page gel this protein migrates as a 160-kDa protein, hence it was initially named p160. In early studies the PELP1 and the MNAR (modulator of non-genomic actions of estrogen receptor) were described as two different proteins; later they were identified as the same substance. PELP1/MNAR contains several motifs and domains that are commonly present in many transcriptional coactivators. The N-terminal domain of PELP1 contain LXXLL motifs which were found to interact with the AF2 domain of ER in a ligand-dependent manner [12].

To our knowledge, the presence of PELP1 in muscle cells of scoliotic patients has never been studied before. That is why the purpose of the study was to check whether PELP1 can be identified in back muscle tissue both at the protein and mRNA level. In this letter, we evaluated the expression and possible localization of PELP1 by immunochemistry, western blot and reverse transcription followed by quantitative polymerase chain reaction (RT-qPCR).

## Material and Methods

We examined 29 girls with idiopathic scoliosis who underwent posterior spinal surgery (Table 1). The age at surgery ranged from 11 years and 10 months to 21 years, mean 15 years and 5 months ± 28 months. The mean angle of the primary thoracic spinal curvature measured on standing antero-posterior radiograph according to Cobb method was 76.1°, range 50° to 114°. Regarding the age of onset, 10 patients presented adolescent idiopathic scoliosis (AIS) while 19 were diagnosed as early onset idiopathic scoliosis (EOIS). According to Lenke scoliosis classification [16] the patients demonstrated four types as follows, type 1: 17 patients, type 2: 2, type 3: 8, type 4: 2 patients. Lonstein and Carlson progression risk factor [17] ranged from 1.9 to 8.5; mean 4.8 ± 1.7, indicating high risk of progression of scoliosis.

**Table 1:** Characteristics of the patients.

Patient	Age at the surgery in months	Type of the scoliosis	Cobb angle	Lenke classification	Progression risk factor
1	237	AIS	52	1	1.9
2	187	EOIS	70	6	3.9
3	238	AIS	62	3	2.5
4	168	EOIS	95	1	5.9
5	180	EOIS	86	1	4.9
6	178	EOIS	80	1	4.9
7	185	EOIS	91	3	5.3
8	161	EOIS	95	3	6.6
9	254	AIS	55	1	1.9
10	142	EOIS	84	1	7.4
11	195	EOIS	96	1	5.2
12	168	AIS	50	1	2.9
13	224	AIS	52	1	2.1
14	200	AIS	52	1	2.5
15	193	AIS	80	1	4.2
16	158	EOIS	55	1	3.8
17	177	AIS	78	3	4.9
18	175	EOIS	84	3	5.1
19	156	EOIS	110	1	7.8
20	195	EOIS	110	3	6.1
21	178	EOIS	64	1	3.9
22	177	EOIS	65	6	3.8
23	164	EOIS	114	1	8.5
24	157	EOIS	70	2	4.9
25	155	EOIS	74	3	5.7
26	164	EOIS	75	1	4.8
27	226	AIS	85	3	4.1
28	176	EOIS	54	1	3.0
29	209	AIS	69	2	3.3

AIS- adolescent idiopathic scoliosis; EOIS- early onset idiopathic scoliosis

Muscle samples (about 1 cm³ each) were harvested from: (1) superficial (trapezius) and (2) deep paravertebral (longissimus) muscles at both sides of the spine at the apical area of the primary thoracic curvature. The material was divided into three parts. Tissue for qPCR was placed in the RNAase protective medium (RNA*Later*; Sigma Aldrich, USA) and stored at -80°C. Material for the immunoelectrophoretic study was placed in phosphate buffered saline buffer (pH 7.5) and stored in -80°C until further analyzes. The tissue for immunohistochemistry was fixed in 4% paraformaldehyde (Chempur, Poland).

The Institutional Review Board at the Poznan University of Medical Sciences approved the study (No 87/09). Prior to their inclusion all patients gave their informed consent, as did the parents.

### Quantitative *PELP1*, *ESR1* and *ESR2* PCR expression analysis

#### RNA isolation and cDNA synthesis

Total RNA was isolated from muscle tissue using TriPure Isolation Reagent (Roche Diagnostic GmbH, Germany) in compliance with manufacturer’s protocol. The quality of total RNA and its concentration were analyzed with use of NanoDrop ND-1000 spectrophotometer (Thermo Fisher Scientific, USA). The integrity was checked electrophoretically in 0.8% denaturing agarose gel in 1xFA buffer (20 mM 3-[N-morpholino]-propanesulfonic acid (MOPS) (free acid), 5 mM sodium acetate, 1 mM EDTA, pH 7.0, Sigma) in presence of ethidium bromide by analyzing the ribosomal RNA bands.

The reaction of reverse transcription was conducted in compliance with Transcriptor Reverse Transcriptase manufacturer’s protocol (Roche) in the total volume of 10 μL. Two step reaction was applied. In the first step mixture of: 5 mmol/L oligo(d)T_10_ (Genomed, Poland), RNA (0.5 μg) and RNase, DNase and pyrogen free water (Life Science) was denaturated 10 min at 65°C. After that the samples were cooled on ice. Second step involved adding and incubation of alternate reagents such as 1U ribonuclease inhibitor (RNasin, Roche), 1U of transcriptor reverse transcriptase (Roche), 100 mmol/L dNTPs (Novayzm, Poland) and 1x reaction buffer (Roche). Thermal profile included: 10 minutes at 25°C (binding of primers to the template), 60 minutes at 55°C (polymerization of cDNA strand) and 5 minutes at 85°C (enzyme denaturation). Until further analysis cDNA was stored in -20°C, but no longer than one week.

#### Quantitative PCR

Obtained cDNA was used for quantitative polymerase chain reaction. The LightCycler 2.0 carousel glass capillary based system (Roche) was applied to RNA expression level analyses. In the case of *PELP1* (GeneBank: NM_014389.2) and all splice variants of *ESR1* (GenBank. NM_000125.3; NM_001122740.1; NM_001122741.1; NM_001122742.1) the qPCR was conducted with TaqMan® Probes (Roche). The probe assays were designed with the ProbeFinder software (http://qpcr.probefinder.com). Because of the low efficiency reaction for splice variants of *ESR2* (GeneBank: NM_001437; NM_001040275; NM_001040276) with use of fluorescent labelled probes we decided to apply the SybrGreen I for the measurement. The primer set in this case was designed using Primer3Plus software ver. 2.3.6 (http://primer3plus.com). Human ready to use *HPRT* Reference Gene Assay (Roche assay N° 05046157001) was used as internal control and for relative concentration ratio assessment.

For *PELP1*, *ESR1* and *HPRT* final concentration in the total volume of 20 μL reaction mix was as follows: 1x Light Cycler FastStart TaqMan Probe Master (Roche), 2.5 μL cDNA, 0.5 mmol/L of each primer pare (Genomed) and 0.1 mmol/L hydrolysis probes (Roche). Gene of interest (GOI) sense and antisense primers and the numbers of UPL probes were: *PELP1* 5’-GCACTGTGTGTCTTGGCTTC-3’ and 5’-GAGGAGGTCCCTCAGGACA-3’ probe #62; *ESR1* 5’-AATGCTACGAAGTGGGAATGAT-3’ and 5’-CAAAGGTTGGCAGCTCTCAT-3’ probe # 67. For *ESR2* in the reaction mix contained: 1x Light Cycler FastStart DNA Master SybrGreen I (Roche), 1.5 μL cDNA, 0.5 mmol/L of each primer (Genomed). The primer’s sequences were: 5’-GAAGCATTCAAGGACATAATG-3’ and 5’-TCCCACTTCGTAACACTTC-3’. The standard curves were constructed using decimal dilutions from 1 to 1:100 000 of RT-PCR products and the reaction efficiencies were calculated for each GOI and reference separately. Randomly selected 10% of samples were sequenced in order to confirm their identity.

Quantitative raw data (threshold values–Ct) were analyzed by comparing it to appropriately selected standard curves and reference gene assay with use of LC 4.05 software and presented as concentration ratio.

The consensus sequence of the qPCR product was confirmed by sanger sequencing analysis and compared to the mRNA of Homo sapiens proline, glutamate and leucine rich protein 1 (PELP1), transcript variant 1, mRNA (GenBank N°: NM_014389.2).

### PELP1 protein expression analysis

#### Protein isolation

Frozen tissue specimens were homogenized in liquid nitrogen and suspended in isolation buffer containing: 320 mmol/L saccharose; 10 mmol/L Tris; 1 mmol/L Mg^2+^; 2 mmol/L DDT; 0.2 mmol/L PMSF; pH 7.5 (Sigma). Then, samples were centrifuged (10 min, 3000g, 4°C). The cell debris pellet was removed and supernatant containing protein extracts was used for further analyses. Protein concentration was measured spectrophotometrically twice for each sample using Quick Start Bradford Dye Reagent (BioRad, UK) according to the manufacturer’s protocol. Standard curve was constructed by measurement of bovine serum albumin fraction V (BSA) (Roche) with decreasing decimal concentrations in the range of 1 mg/mL– 0.001 mg/mL.

#### Western blot analysis

PELP1 protein analysis was done using SDS-PAGE followed by western blot. 30μg of extracted proteins were separated in 12% polyacrylamide gel in denaturating conditions (200V, 40 min, RT). The separation of proteins was observed with the use of prestained protein ladder (PageRuler^TM^ Plus, Part No. 26619, 26620, Thermo Scientific, USA). Proteins were transferred in transfer buffer (300 mA, 90 min), on activated Hydrobond-C Extra membrane (GE Healthcare Europe, France) and incubated with gentle shaking in blocking solution (5% Blotto Reagent, BioRad) for 1 hour, 4°C in TBST buffer (50 mmol/L Tris, 150 mmol/L NaCl, 0.1% Tween 20, pH 7.5; LabEmpire, Poland). Primary polyclonal rabbit antibody (Bethyl Laboratories) against PELP1 and secondary polyclonal goat anti-rabbit linked with EZ-ECL Chemiluminescence Detection Kit (Biological Industries Israel Beit Haemek LTD, Israel) for horseradish peroxidase (HRP) were used in dilution 1:2500. Incubation with each antibody lasted 1 hour at room temperature. Between incubations with antibodies the membrane was washed three times in TBST buffer. The membranes were analyzed and digitized in the context of optical density with use of G:BOX Chemi system (Syngene, UK). Western blotting GAPDH glyceraldehyde-3-phosphate dehydrogenase (GAPDH) protein expression was used as internal control. Totally, 6 patients (20% of the whole group), randomly chosen, were tested with western blot.

#### Immunohistochemistry

Muscle tissue samples underwent the standard procedure of fixation and embedding in paraffin blocks, which were later sectioned with use of microtome. Tissue sections (3 μm) were placed on slides and boiled twice for 10 min in citrate buffer (100 mmol/L sodium citrate solution, 100 mmol/L citric acid solution, pH 6.0, LabEmpire) in a microwave oven (200 W). This allowed the antigen to be exposed. Later, the samples were cooled down at room temperature. The next step was to incubate the slides in 3% solution of H_2_O_2_ (Chempur) for 3 min. in order to block the endogenous peroxidase. Later on, the slides underwent 1 hour incubation at room temperature in blocking buffer (100 mmol/L Tris, 65 mmol/L NaCl, 0,1% Tween 20; 3% BSA, pH 7,5). Expression of PELP1 protein was evaluated using primary antibody (dilution 1:50, Bethyl Laboratories Inc., USA) and the incubation lasted 12 hours at 4°C. Slides were later rinsed using TBS buffer (3x15 min.). After that, the identification of antigen was available with use of Dako EnVision + System HRP (DAB) (Dako Denmark A/S, Denmark) kit according to protocol given by manufacturer. As a positive controls sections of breast and ovarian cancer, incubated with both types of antibodies, were used. Microscopic evaluation was conducted with Axiophot (Zeiss, Germany) microscope. In all samples the regular hematoxylin–eosin staining was performed and studied prior to immunohistochemistry analysis.

### Statistical analysis

The analysis was performed using Statistica 10 software (StatSoft Inc., Tulsa, OK, USA). Nonparametric Kruskal-Wallis test with Dunn’s post hoc test and Spearman rang correlation test were applied. Data were presented as mean ± SD and considered statistically significant at P< 0.05.

## Results

### Presence of *PELP1* gene transcript

**Fig 1:**
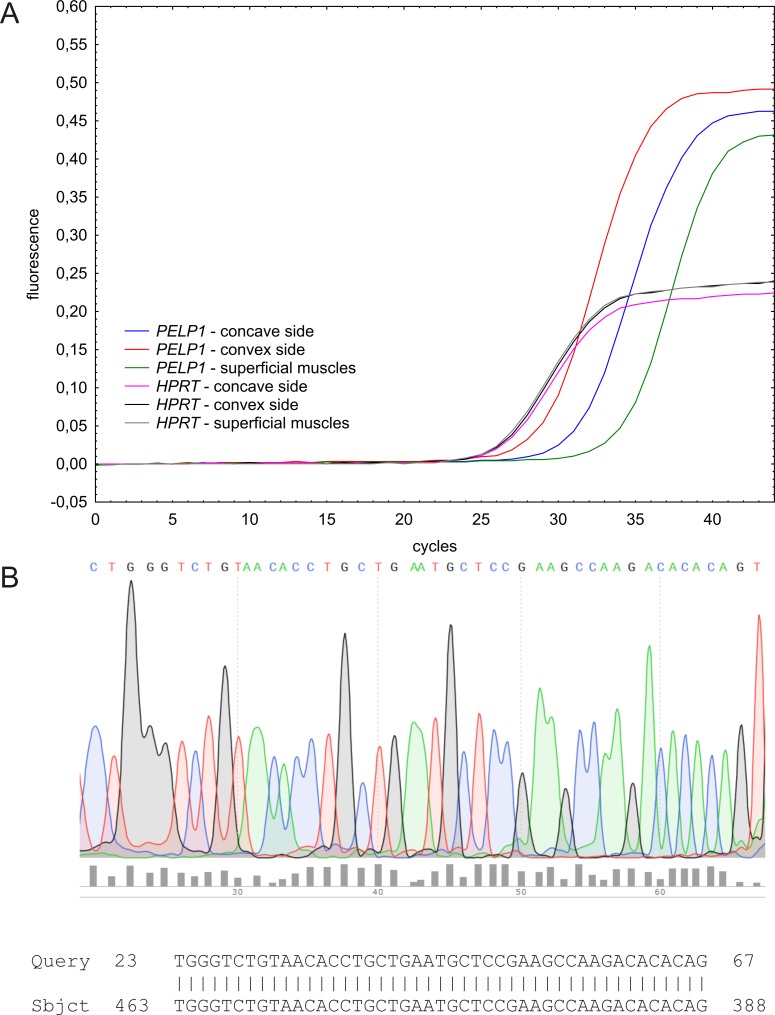
Fluorescence acquisition data. (A) RT-qPCR fluorescence amplification curves of *PELP1* transcript in superficial and deep paravertebral muscles; *HPRT* gene was used as internal control and for relative concentration ratio assessment. (B) Sanger sequencing analysis of the *PELP1* qPCR products in paravertebral muscles samples.

Presence of *PELP1* gene transcript was confirmed using qPCR in all tissue samples, both in deep paravertebral and in superficial back muscles (Fig 1A). 10% of the randomly selected samples were sequenced and they corresponded to the length of consensus sequence of the human *PELP1* transcript variant 1 (Fig 1B).

### Relative expression of *PELP1* gene transcript

**Fig 2:**
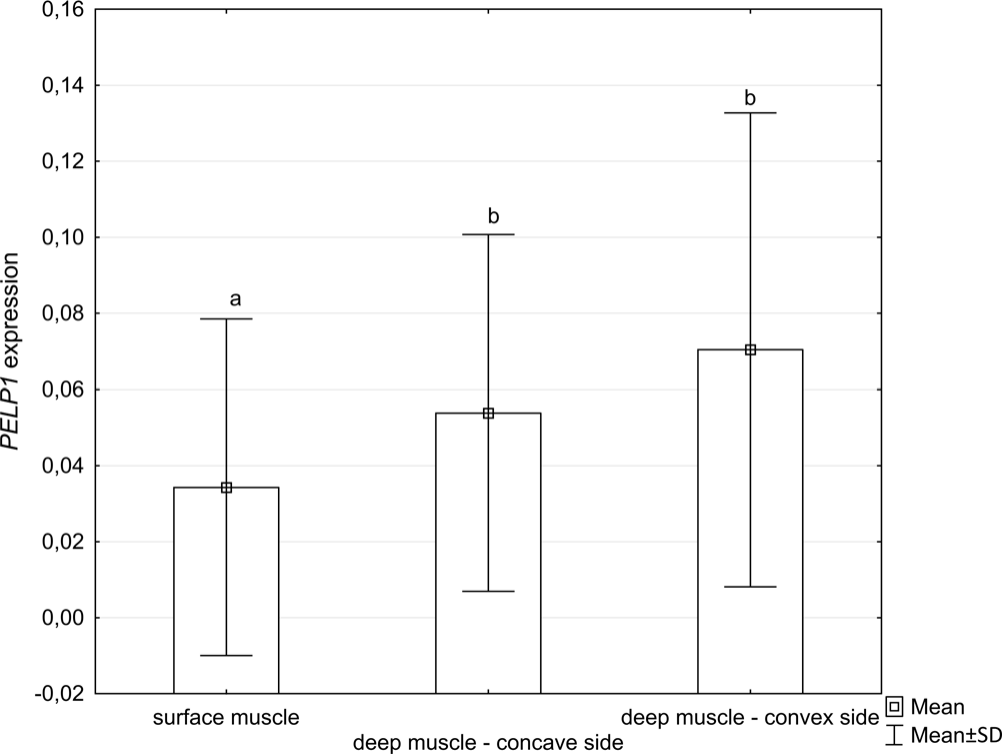
Relative *PELP1* expression level in deep paravertebral muscles and superficial muscles. Different superscript letters above each bar are significantly different, P<0.05 (Kruskal-Wallis test with Dunn’s post hoc test).

**Fig 3:**
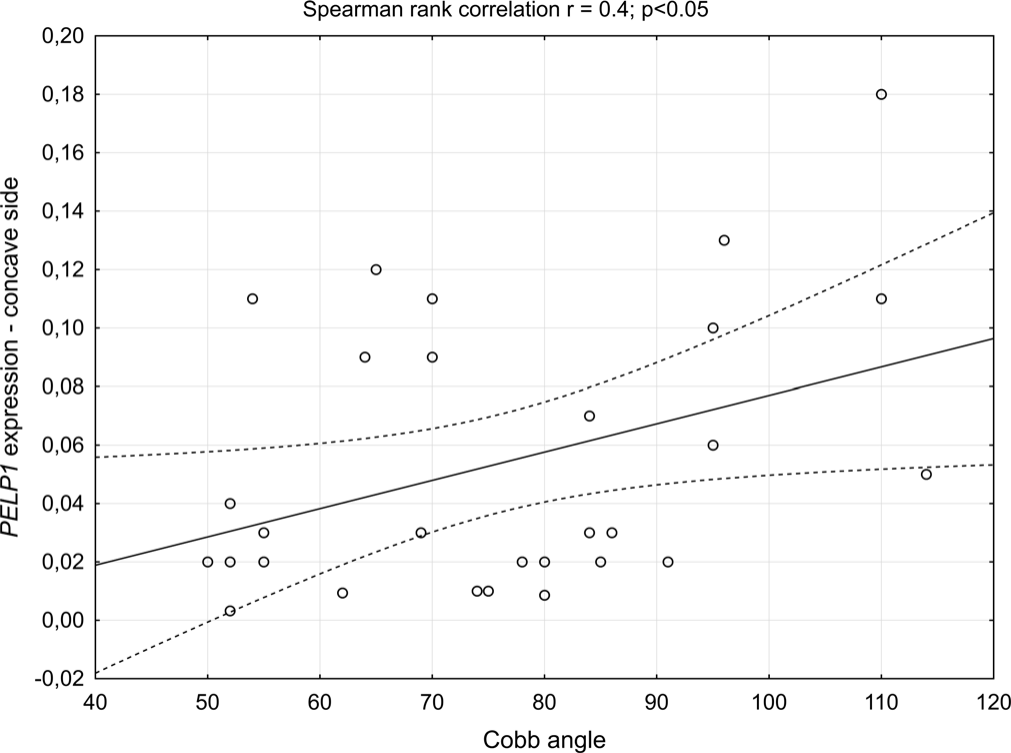
Spearman rank correlation. Correlation between *PELP1* expression level and the Cobb angle on the concave side.

The level of *PELP1* expression in deep paravertebral muscles was significantly higher than in superficial muscles (p = 0.005), Fig 2. Within the deep muscles, although the expression level at the convexity was higher than at the concavity of the thoracic scoliosis, the difference revealed not significant (p>0.05). A moderate correlation between *PELP1* expression level on the concave side and the Cobb angle (r = 0.4; p<0.05) (Fig 3) was noted, while it was not significant for the convex side of thoracic scoliosis (r = 0.4; p>0.056).

### Expression of *PELP1* versus *ESR1* and *ESR2*

**Fig 4:**
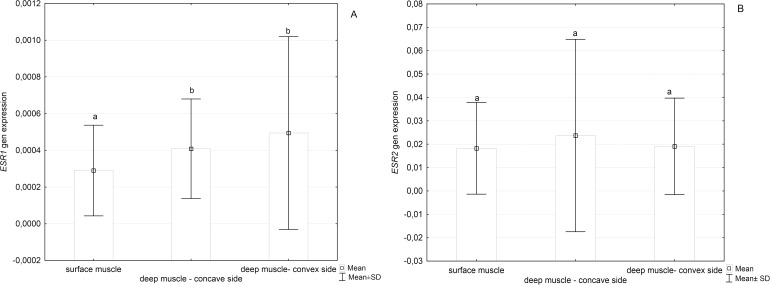
**Relative *ESR1* (A) and *ESR2* (B) expression level in deep paravertebral muscles and superficial muscles**. Different superscript letters above each bar denote significant difference, P < 0.05 (Kruskal-Wallis test with Dunn’s post hoc test).

The level of *ESR1* expression in deep paravertebral muscles was significantly higher than in back superficial muscles (p = 0.01), Fig 4A. No significant differences in the relative level of *ESR2* expression between deep paravertebral muscles and back superficial muscles was observed (p>0.05), Fig 4B. In deep paraspinal muscles, a good correlation between *PELP1* expression level and *ESR1* expression level was seen at the convex side of the curve (r = 0.7; p<0.05) while moderate correlation at the concave side (r = 0.4; p<0.05). No correlation between *PELP1* expression level and *ESR2* expression level at any side of scoliotic spine curvature was found (p>0.05).

### Expression and cellular localization of PELP1 protein

Both the immunohistochemistry and western blot techniques confirmed the presence of the PELP1 protein in paraspinal muscles.

**Fig 5:**
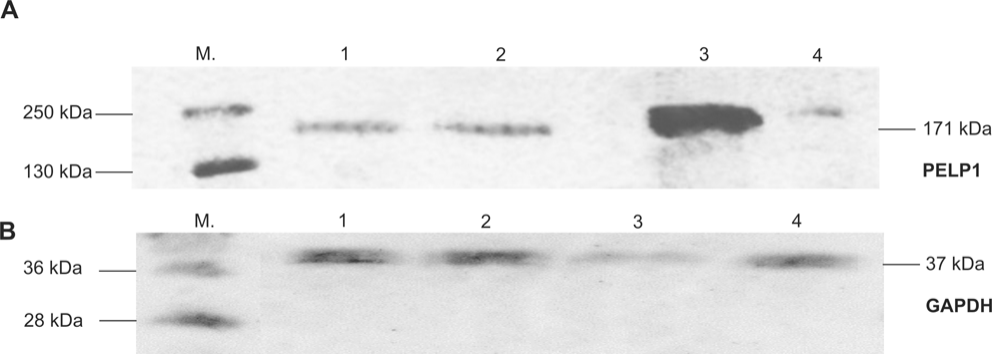
**Western blotting PELP1 protein expression in paravertebral muscles (A)**. Ovarian cancer was used as an external control. Western blotting GAPDH (Glyceraldehyde-3-phosphate dehydrogenase) protein expression was used as internal control (B). Designations: 1 –Page Ruler^TM^ protein ladder, 2 –deep paravertebral muscles concave side, 2 –deep paravertebral muscles convex side, 3 –control (ovarian cancer), 4 –superficial muscles.

Western blot analyses confirmed the presence of the PELP1 protein in all samples tested (. Bands were observed at about 171 kDa. PELP1 protein level was significantly higher in control sample (ovarian cancer). No significant difference between convex versus concave longissimus muscle was observed. The superficial trapezius muscle revealed lower expression of the protein comparing to both deep muscles samples (Fig 5).

**Fig 6:**
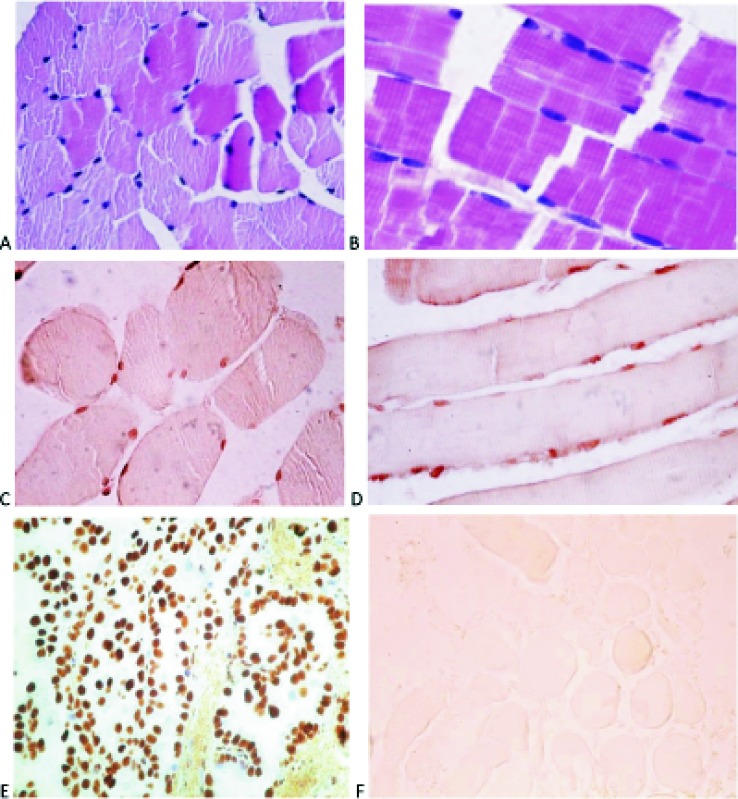
Immunohistochemical localization of PELP1 protein expression in paravertebral muscles. (A, B) Light microscopic images with regular hematoxylin and eosin staining were applied to assess the tissue morphology. The transverse section (C) and the longitudinal section (D) of the deep paravertebral muscle with positive reaction to PELP1 within the nucleus. Control reaction: (E) positive control–ovarian cancer, (F) negative control–paravertebral muscles incubation without primary antibodies.

Positive immunohistochemical staining for PELP1 protein was detected in the nuclei of the muscles cells (Fig 6).

## Discussion

During posterior scoliosis surgery the exposure of the bony elements comprises the dissection of the deep paraspinal muscles covering the vertebrae and is the first stage of operation. After the correction of the curve, during the closure of the wound, some muscle fragments are routinely removed. Thus, by harvesting muscle samples, no additional harm to patient was produced. It is assumed that idiopathic scoliosis, consisting of a sequence of displaced and deformed vertebrae, represents a final consequence of an unknown intrinsic mechanism influencing the vertebrae position and shape. One of probable chain link within this complex mechanism could be localized in deep paravertebral muscles because their activity directly affects the vertebrae by muscular insertions and attachments. As idiopathic scoliosis is a well-recognized three-dimensional spinal deformity, any imbalance in the paraspinal muscles function can be debated as possible factor impairing the physiological shape of the spine. Such imbalance could result from many causes such as neurological stimuli, mechanical factors, or eventually, could represent an effect of muscular imbalance raising from intrinsic cellular modifications of metabolic pathways. Thus, the role of PELP1 in muscular cells of patients with scoliosis deserves deeper analysis.

Recent studies have identified a novel factor, PELP1, described as coactivator of the estrogen signaling pathway. Initially, PELP1 was studied in oncology by discussing its potential proto-oncogene role in the development of hormone-responsive breast cancers [18]. Then, further findings on PELP1 involvement followed, for example in 2013, Yan et al observed it in the periodontal ligament progenitor cells differentiation induced with vitamin C [19]. Yet, the precise place of PELP1 within the signaling chain is still debated. In 2014, Hussey et al. reported on PELP1 involvement in target gene expression acting via histone variant macroH2A1 [20], placing this mechanism into the epigenetic context.

Intracellular pathways of signaling acting via estrogen receptors were reported to be associated with idiopathic scoliosis [21,22]. In 2012, Peng et al. described the possible role of G-protein ESR1 in patients with idiopathic scoliosis by demonstrating association of gene polymorphisms with severity of the curve [23]. We have already published on ESR1 and ESR2 gene polymorphisms association with idiopathic scoliosis occurrence and severity [24,25]. However, in replication studies we could not confirm previously described association of *ESR1* XbaI, *ESR1* PvuII or *ESR2* AlwNI with IS occurrence or severity. The *ESR2* AluI polymorphism may be associated with IS severity [25].

In this study, we evaluated the recently described factor, PELP1, in the context of its possible involvement in the complex mechanism of onset and progression of spinal curvature within the frame of idiopathic thoracic scoliosis. However, having no control group, we cannot suggest it could play a role in the pathogenesis of IS. We identified the presence of PELP1 at various levels: as expression product of the corresponding gene and as 171 kDa protein detected with western blot technique and visualized in the muscular tissue by immunohistochemistry. This was demonstrated for the first time in the muscles lying along the spine and attached to the thoracic vertebrae and for the first time in patients suffering from thoracic idiopathic scoliosis. We have identified one report on PELP1 expression in skeletal muscles [26] but not in deep paravertebral muscles. Moreover, we analyzed relative expression of *PELP1* and found significantly higher expression level in the deep intrinsic spine muscle (longissimus) comparing to the superficial back muscle (trapezius), the latter belonging to the upper limb girdle muscles. This corresponds with the different role of the two muscle groups, the deep muscles (and not the superficial ones) being indicated as eventually involved in scoliosis pathogenesis. Also, a correlation of *PELP1* expression on the concave side with Cobb angle of the primary thoracic scoliosis was observed. Another interesting point was a good correlation between the *PELP1* and the *ESR1* expression. Such correlation was not observed with the *ESR2*. This observation suggests an important role of PELP1 in the estrogen signal transducing system based on ESR1. PELP1 was shown to interact with nuclear proteins however, it was also described as a scaffolding molecule in the cytoplasm [13]. In this study, we have identified the nuclear location of PELP1 within muscular tissue (Fig 6) which suggests a significant role in chromatin remodeling at target gene promoters.

Interestingly, although the evidence that idiopathic scoliosis progression occurring mostly in girls around puberty has already raised extensive research on estrogen receptors genes polymorphisms and G-protein involvement, the discussion of possible role of estrogens in the development of idiopathic scoliosis was longtime influenced by the ambiguous findings on different estrogens serum levels in adolescent females [8]. However, recently it was demonstrated that systemic serum levels of estrogens cannot be directly considered as intracellular hormone content because the main estrogen, 17-beta-estradiol, was proven to be actively synthetized by muscular cells [27]. Moreover, most recently, in 2015, Pöllänen et. al. reported that the intramuscular estrogens are associated with skeletal muscle strength and power in females [28]. These findings offer new extensions to the discussion on impact of female steroid hormones on muscle function in girls with idiopathic scoliosis.

The limitations of this study comprise the case only pattern without control group which could not be obtained for ethical reasons. Also, the degree of Cobb angle was high up to 114°; it is not clear whether the macroscopically observed differences between the concave and the convex side in severe scoliosis (muscle tissue fibrous and lipid degeneration more expressed at concavity) are or are not mirrored at the cellular level.

## Conclusion

In conclusion, three techniques for the first time demonstrated the presence of the PELP1 in paraspinal muscles of patients with thoracic idiopathic scoliosis. The PELP1 potential regulatory impact on back muscle function is to be further investigated.
